# Routine Family Planning Data in the Low- and Middle-Income Country Context: A Synthesis of Findings From 17 Small Research Grants

**DOI:** 10.9745/GHSP-D-20-00122

**Published:** 2020-12-23

**Authors:** Bridgit Adamou, Janine Barden-O’Fallon, Katie Williams, Amani Selim

**Affiliations:** a University of North Carolina at Chapel Hill, Chapel Hill, NC, USA.; b United States Agency of International Development, Washington, DC, USA.

## Abstract

Health information systems rely on high-quality data to measure, track, and inform decision making. Currently, the quality, uptake, and use of family planning data in routine health information systems is limited, presenting an opportunity for improvement on many levels. The current synthesis assessed findings from 17 small grants that MEASURE Evaluation issued to low- and middle-income country research teams between 2015 and 2019. Main findings from that research were collaboratively categorized in 4 major themes: (1) the enabling environment for managing and using family planning information; (2) barriers to integration of family planning in routine health information systems; (3) gaps in the analysis, interpretation, and use of routine family planning data; and (4) family planning data use in management, programmatic, and budgetary decisions. Data quality at the systemic, organizational, technical, and output levels was a crosscutting theme. Collectively, the findings outline barriers to and opportunities for improved integration of family planning data and subsequent strengthening of routine health information systems.

## Abstract (precis)

A review of 5 years of small grant-funded research highlighted overarching barriers to and opportunities for using family planning data in routine health information systems in low- and middle-income countries. We report on factors affecting data quality, analysis, and use, and suggest strategies to improve routine family planning data.

## Teaser

**Key Findings:**

- We found 4 major themes affecting family planning data quality, analysis, and use:
  - The enabling environment for managing and using family planning information
  - Barriers to integrating family planning in routine health information systems
  - Gaps in analyzing, interpreting, and using routine family planning data
  - Family planning data use in decision making
- Systematic, organizational, cultural, and technical barriers affect data quality and limit subsequent analysis, interpretation, and use of information.

**Key Implications:**

- Program implementers should consider:
  - Standardizing family planning indicators across sectors (public and private) and data collection tools
  - Conducting regular staff trainings and capacity building to improve data literacy, collection, and reporting
  - Investing in the human and technological resources needed for effective data collection, analysis, and use.
- Policy makers should:
  - Enact and commit to continuous financial support
  - Emphasize well-defined data collection and reporting processes, including clearly defined indicators and harmonized data collection tools
  - Provide well-supported technical infrastructure.

## BACKGROUND

The provision of health care services and information about their quality and quantity are critical components of a health system. These components must function together to strengthen service delivery programs and improve population health. Countries use health information systems (HIS) to measure and track health services, allowing them to plan, evaluate, and implement health strategies.^1^ An efficient HIS draws from multiple levels of the health system, using clearly defined indicators, up-to-date standards and guidelines, accessible data collection and analysis tools, and stakeholder collaboration and support to enable evidence-informed decision making.^2^ A key component of an HIS is a routine health information system (RHIS), fundamentally composed of indicators to track management information needs and data collection, transmission, processing, and analysis, which should all lead to information use.^3^ Data from RHISs include service statistics, management and logistics data, and financial data, and provide information on client health status, facility and budgetary capacity, and services and resources administered or available.^4^ These RHIS data constitute the main pillar for monitoring service delivery programs at the national level in low- and middle-income countries (LMICs).^5^ Despite a sound framework for an effective HIS, earlier research found underperforming RHISs due to several factors, such as poor data quality; indicators lacking standardization, clear definitions, and accurate calculations; inadequate electronic data capture and reporting; incomplete data analysis; poor management support; and weak use of information for planning and decision making.^4^ A strong RHIS that supports data-informed decisions requires 4 key actions: regularly assessing the organizational, technical, and behavioral factors that affect decision making to improve data demand and use; engaging data producers (those who design and manage research and information systems) and data users (those who use data in program improvement and development) in the decision-making cycle; improving data quality; and improving data availability, defined as data synthesis, data communication, and access to data.^6^

For many LMICs, accurate collection, reporting, analysis, and use of routine data from an HIS are challenging tasks that span health areas, from maternal and child health to infectious and chronic diseases.^7^ It is also a challenge for LMICs to ensure that routine family planning data in their HISs are accurate and complete. The family planning community has paid relatively little attention to strengthening RHISs, causing the field to fall behind other health areas.^8^ Recent efforts to collect data for the FP2020 global initiative have brought increased attention to family planning service statistics, data quality, and reporting mechanisms.^8^

Despite the recent attention focused on family planning in RHISs, the production of high-quality information sufficient for program planning, monitoring, advocacy, and other decision-making needs has proven difficult. Health care providers that do collect routine family planning data often find that the larger HIS into which these data feed lacks the appropriate reporting or synthesis mechanisms; in other cases, the family planning data are of poor quality or are not collected consistently.^9^ Knowledge gaps related to routine family planning data include how to improve the quality of family planning data, how to address barriers to integrating family planning data in RHIS, and how to encourage analysis and use of the data to improve family planning outcomes.

To better understand the dynamics of family planning data collection, integration, and use, the MEASURE Evaluation project, funded by the U.S. Agency for International Development (USAID), provided technical and financial support for researchers in LMICs to investigate issues related to the collection, aggregation, and use of routine family planning data. This article synthesizes the family planning-specific research results from 17 small grant-funded projects, organized by common themes, to shed light on the status of family planning in RHISs.

This synthesis of family planning–specific research results from 17 small grant–funded projects sheds light on the status of family planning in RHISs.

## METHODS

In 2014, MEASURE Evaluation implemented a program funded by the USAID Office of Population and Reproductive Health that provided small grants for research related to the collection, analysis, and use of routine family planning data in 24 priority countries. The overarching goal of the program was to produce evidence that could help improve RHISs and advance family planning outcomes. The MEASURE Evaluation small grants program aimed to (1) address research gaps in routine health information for family planning/reproductive health (RH) to inform policy and programmatic decision making, (2) strengthen research capacity among local agencies, and (3) increase use of research findings by providing an opportunity for the data to be disseminated to and used by local stakeholders to inform decision making. The program supported both primary and secondary data collection and analysis. Grant recipients were required to secure appropriate ethical review and approval prior to research implementation. Five rounds of awards were implemented over a 5-year period (2015–2019), generating 360 applications and resulting in 19 funded research projects in 11 countries (Table). Recipients represented a mix of university, quasi-governmental, nonprofit, and private research organizations. The grant amounts ranged from US$10,000 to US$24,000 in direct funds, with an average award of US$14,400. We required recipients to complete a technical working paper of their research results and to conduct at least 1 data use activity with stakeholders (such as the presentation of findings at technical working group meetings, workshops, or conferences). We provided technical assistance as needed throughout the application, implementation, writing, and dissemination stages of the research projects. Details about the program were previously described by Adamou.^10^

**TABLE.** Seventeen Recipients of Small Grants Funded by MEASURE Evaluation Phase IV, 2015–2019

**Research Organization**	**Research Title**	**Study Objective(s)**	**Geographic Coverage**	**Data Sources**
Integrated Health Initiative	Integrating Family Planning Data from Public and Private Health Facilities in Malawi: How Current Approaches Align with FP2020 Goals^12^	Find approaches to improve the national Health Information System by integrating family planning data from private-sector service delivery points and government facilities	2 districts in each of the 3 regions in Malawi	Desk reviews of all national policy documents guiding family planning data and data collection; field observations; 71 KIIs with staff from national-level institutions of the MOH, zonal offices of the 5 quality control divisions (i.e., zones) in Malawi, and family planning service providers, HMIS officers, health surveillance assistants, family planning coordinators, and data clerks
Rivers State of Nigeria Primary Health Care Management Board	Use of Technology to Manage Health Data in Rivers State, Nigeria: A Qualitative Study on Family Planning and Routine Health Information Systems^13^	Explore the experiences and perceptions of family planning providers and health information officers on implementing technology for district health data collection and identify factors that affect the sustainability of using technology for data management in Rivers State, Nigeria	Rivers State, Nigeria	21 IDIs with state- and LGA-level HMIS officers, desk officers, monitoring and evaluation officers, and reproductive health coordinators; 2 FGDs with 35 facility health information officers and family planning providers
Africa Field Epidemiologic Network	Family Planning Indicators Assessment and Data Quality Audit in Selected Health Facilities Across Nigeria^14^	Estimate family planning indicator performance at the health facility level from the HMIS not reflected in DHIS2 to determine the quality of family planning data at the facility level and identify challenges to family planning program implementation in sampled health facilities in Nigeria	2 LGAs in each of the following 6 states in Nigeria: Bauchi, Delta, Enugu, Kano, Osun, and Nasarawa	Administration of a questionnaire via interviews with 114 family planning/reproductive health focal people in selected facilities; 42 KIIs with family planning stakeholders and key decision makers in the family planning/reproductive health units at the LGA and state levels in the selected states; 6 FGDs with health workers/service providers
The Rescue Initiative-South Sudan	Analyzing, Interpreting, and Communicating Routine Family Planning Data in South Sudan^15^	Explore how effectively family planning data in the RHIS are analyzed, interpreted, and communicated, and discuss barriers to RHIS data use and ownership in 2 states in South Sudan	17 counties in 2 states in South Sudan: Central Equatoria and Western Equatoria	Direct observation at service delivery points, individual questionnaires administered to health facility staff, and KIIs with a total of 180 study participants
University of the Punjab, Institute of Social and Cultural Studies	The Routine Health Information Systems in Punjab Province, Pakistan: Exploring the Potential for Integrating Health Information Systems for Family Planning Data^16^	Review the RHIS in Punjab province of Pakistan and explore the potential for integrating community-level data into the national HMIS, particularly family planning data, collected by public or private, for-profit, and not-for-profit organizations	Punjab province, Pakistan	Document review and 16 KIIs with lady health workers, the Population Welfare Dept., Rahnuma–Family Planning Association of Pakistan, DHIS office, United Nations Population Fund, and United Nations Children’s Fund
Department of Population Studies, Makerere University	Integrating Family Planning Data in Uganda’s Health Management Information System^17^	Investigate the facilitators, best practices, and barriers of integrating family planning data into the district and national HMIS in Uganda	Kampala, Jinja, and Hoima districts, Uganda	16 KIIs with MOH officers, HMIS focal persons at nongovernmental organizations, HMIS focal persons who were district biostatisticians or medical records officers, and providers who were medical records officers at public and private health facilities; a multi-stakeholder dialogue workshop comprised of 11 participants; and a systematic review of the HMIS in sub-Saharan African countries that are United States Agency for International Development family planning priorities
International Centre for Diarrhoeal Disease Research, Bangladesh	Using DHIS 2 Software to Collect Health Data in Bangladesh^18^	Explore the perceptions and experiences with using DHIS2 to collect and analyze reproductive, newborn, maternal, and child health data in Bangladesh and to identify facilitators and barriers to using these data at different levels of the health care system	Khulna and Chittagong districts in Bangladesh	Document review; 23 IDIs with community health care providers, nurses, health inspectors, and upazila statisticians; 2 FGDs with district statisticians; and 11 KIIs with health managers, HMIS experts, and key decision makers
Research and Development Division, Ghana Health Service	Experiences and Perceptions of Health Staff on Applying Information Technology for District Health Data Management in Ghana^19^	Explore and document the experiences and perspectives of health staff and managers in the 4 districts on use of mobile technology to collect and manage health data in district health systems	4 administrative districts in Ghana’s Central Region	KIIs with 160 frontline health staff (midwives, community health nurses, health information officers, general nurses, and physician assistants) at both the district and subdistrict levels and 14 district and regional health managers and policy makers
Centre of Population, Health and Nutrition Services	Improving Family Planning Service Delivery in Ghana^20^	Map out the distribution of all family planning service providers in the region and document how the community-based family planning information system is linked to the national system to recommend strategies for supporting program planning and implementation and improving family planning services	Upper East Region, Ghana	Records review and data extraction from DHIS2; survey of all types of service providers in the region’s 13 districts by interviewing the family planning providers present (435) using a structured interview questionnaire; 2 FGDs with the district health management team, staff from different subdistrict health teams, and community health officers
Governance Links Tanzania	Strengthening Tanzania’s Routine Health Information System: Incorporating Family Planning Quality Assessment Indicators^21^	Investigating the benefit of incorporating indicators related to family planning quality assessment in a decentralized RHIS in rural farming districts around Lake Victoria	Administrative district of Magu, Mwanza Region, in the Lake Victoria zone of Tanzania	Literature review; questionnaire-facilitated individual interviews with 50 health service providers and community health workers; 12 KIIs with health service providers, pharmacy staff, civil society organization staff, council health management team members, and district health information officers; 2 FGDs with 40 health service providers and community health workers
Matibabu Foundation	Integrating Family Planning Data in Kenya’s DHIS 2^22^	Investigate integration of family planning data in DHIS2, the factors related to lack of integration, and ways to remedy the lack of integration	Siaya and Nairobi counties, with a pretest conducted in Kisumu county in Kenya	Eight KIIs with MOH officers from Siaya and Kisumu counties and a representative from the Division of Health Information Systems, at the national level. Four FGDs were conducted with clinicians, nurses, health records officers, and information officers from both public and private health facilities at all levels, from the primary level to county referral hospitals.
Equitable Health Access Initiative	The Strongest Motivators for Using Routine Health Information in Family Planning: A Prospective Study in Lagos, Nigeria^23^	Bridge the knowledge gap concerning the motivators behind using routine health information in family planning to improve the use of family planning services	3 LGAs of Lagos state, Nigeria	12 KIIs and 425 questionnaires with men and women working in the health sector
Afya Research Africa	Family Planning Services in Kenya During a Transition: Utilization Trends Across Counties^24^	Estimate the general prevalence of family planning use among women of childbearing age and the prevalence of family planning use by county; analyze the trends in family planning utilization over the period of transition, from 2012 to 2015; and estimate the extent to which counties had integrated reporting of family planning services in Kenya’s DHIS2	Kenya	National family planning–related DHIS2 data and Kenya Demographic and Health Survey 2014 data
Mzumbe University, School of Public Administration and Management	Creating a Culture of Data Use in Tanzania: Assessing Health Providers’ Capacity to Analyze and Use Family Planning Data^25^	Understand health providers’ capacity to analyze collected family planning data and to document available evidence of health service providers using the collected data in theirplanning processes	2 LGAs within each of the following regions in Tanzania: Lindia, Geita, and Arusha	13 IDIs with facility in-charges, reproductive and child health in-charges, data clerks, and family planning facility-based providers; 2 FGDs with 24 health providers; and non-participant observation in 12 health facilities
Health Promotion Tanzania	Enhancing Use of Routine Health Information for Family Planning to Influence Decision Making in Tanzania^26^	Explore the type of family planning information collected, how the data are analyzed, and how the information informs planning and budgeting. It examined ways data are handled across all 5 levels of the health system (i.e., national, regional, district, ward, and village) and when and how the data are utilized.	Kilimanjaro and Mara regions of Tanzania	31 KIIs with health officers in charge, points of contact for family planning or reproductive health and child health, district medicalofficers, health governance committee, HMIS focal people, and health secretaries from a regional hospital, district hospital, health center (ward level), and dispensary (village level)
Association for Reproductive and Family Health	Use of Routine Health Information to Inform Budgetary Allocation for Reproductive Health in Cross River State, Nigeria^27^	To understand the budget process within the state MOH and in the health department of the Calabar municipal local government council; examine the use of routine health information as evidence for budgetary allocation for reproductive health and family planning; identify barriers and constraints to routine data use; explore possible solutions; and dialogue with the stakeholders on how routine health data can be used in the budget process	Calabar Municipal LGA in Cross River state, Nigeria	Desk review of existing family planning data in Cross River State and Calabar Municipal LGA, KIIs with staff from relevant ministries, and questionnaires administered to middle- and junior-level officers at the state and LGA levels
Access Global Ltd.	Uganda’s Resources to Finance Family Planning Commodities: Implications for a Total Market Approach^29^	Understand the extent to which in-country resources can mitigate financing shortages for family planning commodities in Uganda, and the implications of a total market approach	Uganda	Literature review; retail audits in 16 pharmacies in Mukono district; and 6 researcher-administered questionnaires with family planning program managers

Abbreviations: DHIS, District Health Information Software; FGD, focus group discussion; HMIS, health management information system; IDI, in-depth interview; KII, key informant interview; LGA, local government area; MOH, ministry of health; RHIS, routine health information system.

To synthesize the results of these research projects, we reviewed the 19 small grants working papers, excluding 2 from the synthesis because the research topics were not specifically related to an RHIS. The main findings of the 17 remaining papers were extracted, reviewed, and organized by key concepts through an iterative process in which all co-authors participated. Themes were developed around the key concepts. Once organized, the findings within each theme were compared and contrasted. We then summarized the results to present main findings for each theme and to contribute to an overall understanding of current strengths, issues, and gaps in family planning data and RHISs in LMICs.

## RESULTS

The synthesis of results yielded the following main themes: (1) the enabling environment for managing and using family planning information; (2) barriers to integration of family planning in RHISs; (3) gaps in the analysis, interpretation, and use of routine family planning data; and (4) use of family planning data in management, programmatic, and budgetary decisions. All papers discussed the issue of data quality—the systematic, organizational, cultural, and technical barriers that contributed to data quality problems and the effects of poor data quality on analysis, interpretation, and use of information. For this reason, data quality was considered to be a crosscutting theme, and we incorporated it, as appropriate, in each of the 4 thematic areas.

### Theme 1: The Enabling Environment for Managing and Using Family Planning Information

The first theme identified in the review of the small grant-funded research papers was related to the enabling environment for the management and use of family planning information. We used the following definition for enabling environment: strong HIS governance and leadership; policy and framework compliance; appropriate resources, such as staffing, technology, and tools; and cross-sector engagement of actors, including private and public entities.^11^ The small grant–funded reports illustrated how challenges in the enabling environment affected data collection, assessment, and use at all levels.

#### HIS Governance and Leadership for Compliance

The review indicated that the strength of system governance can be gauged by a country’s ability to enforce its reporting policies and guidelines. Study findings from Malawi, Nigeria, and South Sudan revealed noncompliance and inconsistent submission of family planning data to the national HIS.^12^ ^–^ ^15^ Weak governance structures were reflected by countries’ inability to enforce guidelines. For example, despite the protocol in Malawi that private franchises must submit their monthly data summary reports to the district health office, private providers felt no obligation to do so.^12^ One study participant shared:

*When we have compiled the data each month we have a summary, and that summary is sent to our headquarters. Yeah, that’s all, it’s sent to our headquarters. The government has never asked me; of course, I have never sent them any data, no.* —Private health service provider

The review showed that the strength of system governance can be gauged by a country’s ability to enforce its reporting policies and guidelines.

In Pakistan, several private facilities are not legally registered, so it is difficult to collect routine health information from them.^16^ However, researchers in Uganda found that because the Ugandan Ministry of Health mandates regular submission of HIS reports to health districts as a requirement for private facilities’ renewal of licensure, private and nongovernmental organization health facilities have greater participation in the HIS.^17^ Furthermore, private nonprofit health facilities (such as faith-based health centers) performed better than public facilities with respect to submission of data because of strict rules enforced by their governing institutions.^11^

#### Appropriate Resources

Researchers in Bangladesh identified a shortage of human resources, frequent version changes in the District Health Information Software, version 2 (DHIS2) platform, negative attitudes about electronic data capture systems from some staff, and reliance on donor support as structural barriers to the success of the HIS.^18^ Consequently, users of the system suggested strong government commitment, deployment of data-quality checks, and accessible technology, along with extensive, sustained financial support, to make the nationwide implementation of the electronic system successful.^18^

The review also found that a consistent factor in managing an RHIS and the subsequent enabling environment for family planning information was the use of new HIS technologies as an important resource for data capture and reporting. Although the reports mentioned several types of systems, many of the national HIS included a web-based application for electronic data management that was accessible through electronic devices with browser and Internet access. Typically, this application was DHIS2. Research in Uganda found that DHIS2 was considered appropriate and user friendly, and the web-based reporting eased the sharing of health data with stakeholders.^17^ Researchers in Ghana found that mobile tools enhanced job performance, the quality of data collection, and the efficiency of data management.^19^ A study participant shared the following:

*I can now sit in my office and monitor activities at the peripheries and even at hard-to-reach areas, which activity would otherwise have cost transport, fuel, and much time. Now, I can go on [the mobile technology] and check … everywhere a health facility is located, or a health staff may work with ease using technology.* —District-level health manager

Nevertheless, the implementation of new technology hindered progress when necessary resources and infrastructures were inadequate. For example, one-third of the 435 family planning service delivery points surveyed in the Upper East region of Ghana did not have electricity, making electronic data very challenging.^20^ Research from Rivers State, Nigeria found the new technology led to parallel systems. Health facilities reported family planning data into DHIS2, but system users continued to use paper-based data collection tools at the health facilities because of logistical challenges with the electronic infrastructure including frequent power outages, hardware problems, broken mobile devices, and lack of Internet connectivity.^13^ Nearly all (96.6%) of the study participants in the Central region of Ghana concurrently used paper-based data collection and reporting tools and mobile technology for collecting and transferring health data.^20^ The research teams in Bangladesh and Tanzania found similar barriers.^18^ ^,^ ^21^ Additionally, the researchers in Rivers State, Nigeria reported faulty computer equipment, inadequate training on use of data tools, and low levels of information and communication technology skills.^13^ Study participants complained of substandard government-issued mobile devices and difficulties using mobile phones for data collection^13^:

*Some of us are not so perfect with the phone, because, eh, at our local government area, we find it difficult to send the message on the phone. But when you get to where you can connect to the Internet, they say “no service.” You will continue waiting, waiting, waiting until you are fed up. At the end of the day, the phone itself, which we are given to serve at the health facility, remains faulty. So, it wasn’t so adequate with us.* —Health information officer at public primary health center

The implementation of new technology hindered progress when necessary resources and infrastructures were inadequate.

Another example of inadequate resources to support an enabling environment was insufficient funding to support district health offices. This translated into scarce resources needed for a fully functioning HIS, such as data collection guidelines; computers and mobile devices; paper record books and forms; and HIS staff available for data consolidation, verification, analysis, and supportive supervision.^17^ ^,^ ^20^ ^,^ ^22^ A district-level study participant in Ghana said^20^:

*I am one person in this office who enters reports from all those facilities into the system, who does data assessment, who analyzes, validates, and everything.*

#### Cross-Sector Engagement

The often-dissonant relationship between public and private health care sectors played a large role in stratifying data collection and limiting information sharing. Even public and private service providers who operated in the same data catchment space often used separate protocols, separate planning procedures, and data collection mechanisms that were not standardized.^9^ ^,^ ^16^ ^,^ ^20^ The differing approaches to family planning data collection and reporting weakened data sharing in the absence of collaborative networks. Study respondents in Malawi estimated that less than half of the data generated in the private health facilities were reported.^12^ Although a system existed to flow data from the facility level to the national HIS, major issues with private-sector actors (e.g., noncompliance, inconsistent data submission, poor-quality data, and reporting delays) prevented interpretation of these data.^12^ The study in Pakistan reflected a similar culture of noncompliance and noncooperation.^16^ In contrast to these findings, research in Uganda found that collaborative networks existed between donor-funded implementing partners and local organizations, enabling training, financial support, and technical assistance in designing data collection tools essential for better HIS performance and sustainability.^17^ This was seen as an opportunity to improve public–private facility interaction by strengthening and standardizing reporting requirements.^17^

### Theme 2: Barriers to Integration of Family Planning in RHISs

The second theme that emerged from the review centered on barriers to the integration or inclusion of family planning as a health area in RHISs. Generally, the studies revealed poor data flow from the service delivery points to the district and national HISs; challenges with implementing data collection tools; lack of clear, standardized family planning indicators; and disjunctive networks of collaboration as limitations to the full integration of family planning in RHIS. Many of the studies revealed incomplete integration of family planning data along the designated data-flow chains, and discrepancies existed between mechanisms for data collection and management at the national, community, and facility levels.^17^ ^,^ ^20^ ^,^ ^22^ For example, research in Kenya revealed that the paper-based national data summary tool, known as the MOH 711, which is used as a template to transfer data to DHIS2, includes family planning methods that are not recorded in either family planning registers or DHIS2. A health official in Kenya remarked, “I know there is no specific one [tool] for family planning that is really standard for all.”^22^ This lack of data harmonization creates ambiguities in the system, compromises data quality, and makes the family planning situation incomplete.^23^ ^,^ ^24^ Multiple studies found discrepancies in the ability to collect and record family planning data specifically at the facility or community level.^15^ ^,^ ^20^ ^,^ ^22^ In Ghana, there were no required reporting mechanisms for certain community-level family planning service providers, such as pharmacies and licensed chemical sellers.^19^ Similarly, the HIS in Pakistan does not have a mechanism to record both community- and facility-based family planning services for each client.^16^ Because the country’s management information systems (the DHIS and commodity logistics management information systems) are managed by different departments, integrating the systems will require high-level organizational restructuring.

Studies revealed limitations with integrating family planning in RHIS: poor data flow from service delivery points to district and national HISs and challenges with implementing data collection tools.

As suggested in Theme 1, issues with technical infrastructure, such as mobile and web-reporting challenges, and restricted access to computer-based systems negatively affected data integration and flow.^12^ ^,^ ^17^ ^,^ ^20^ For example, in Kenya, data entry and editing rights are restricted to the subcounty health records and information officers. This restriction hinders service providers’ ability to efficiently and effectively record family planning data, which ultimately affects what is captured in DHIS2.^22^ A study respondent explained the problem:

*The task sometimes overwhelms the staffs, who would end up with forgetfulness. The notion of I’ll tally tomorrow, and again, tomorrow comes—I’ll tally the next day. So, it is continuous. When you come back tallying at the end of the month, you end up tallying wrong information. Your addition might not be right, so you find discrepancies in data. DHIS2 is not the same as data in the facility. This has happened several times. We even have this report last week, during review meeting, and underreporting—to mean what we have on the ground is not what we have at DHIS2. It’s either due to shortage of staffs, or somebody is not able to fill in data at the right time. The ideal is, one should give the service and then tally real time, then give the document by the end of the day tally. —*Facility in-charge at public health facility

Organizational factors, such as a failure to prioritize family planning data, also influenced integration into the RHISs. Research from Pakistan reflected this prioritization problem; although an RHIS existed for various health care entities, public departments and nongovernmental organizations did not regularly report family planning data into it.^16^

Insufficient human resources for both provision of services (and therefore data capture) and supportive supervision and feedback, too few data collection tools (i.e., computers, tablets, forms, and family planning record books), incorrect data entry, and lack of harmonization of data collection tools also affected the inclusion of family planning data into the RHIS. Problems with data collection tools included electronic and paper-based forms without family planning indicators, improper report consolidation, and unavailable collection mechanisms.^16^ ^,^ ^17^ Additionally, many health facilities involved in these studies operated both with paper-based patient registers and electronic systems, and these disjointed methods led to missing or incomplete data entry—a problem that was compounded by a lack of training for data collectors and a lack of supportive supervision.^17^ ^,^ ^20^ ^,^ ^22^ For example, when forms are revised, not all family planning providers are trained on the changes, which exacerbates the problem of low data literacy and results in family planning data being excluded from the HIS. A district-level health officer in Uganda revealed^17^:

*I have never heard of nurses and midwives going for refresher training on family planning data in the HMIS [health management information system].*

Poor integration of family planning data into the RHIS also stemmed from the limited pool of standardized family planning indicators both in health facility registers and the national HIS.^19^ ^,^ ^23^ In Kenya, researchers found that weak indicators at the facility level affected summary data compiled at the intermediary ministerial level, in turn limiting tertiary indicators in the national HIS.^22^ Without well-defined, standardized indicators harmonized across the HIS, the data collection tools fell short in recording family planning practices and services. The study in Pakistan found that this data shortcoming spurred provider dissatisfaction with the existing family planning indicators.^15^ Data collection forms did not provide indicator definitions or a place to record changes in family planning choice by individuals.^15^ Indicator limitations led to such data-quality issues as inaccuracy, overreporting, and missing family planning measurements.^15^

### Theme 3: Gaps in Analysis of Routine Family Planning Data

The third major theme of the review related to gaps in analysis of routine family planning data. All the research papers underscored that problems, or the perception of problems, with data quality and reliability resulted in limited analysis and use of routine family planning data. For example, Tanzanian researchers found that more than 90% of their study respondents agreed that a big limitation in assessing routine family planning data was poor-quality data (another being the lack of financial resources to support the collection of high-quality routine data).^23^ The limited analysis of routine data was also mentioned as a result of a lack of training on electronic data capture tools, a lack of data literacy among system users, poor data analysis skills, overburdened human resources, and an absence of leadership or guidance for family planning data analysis.^21^ ^,^ ^25^ ^–^ ^27^ The researchers found that there was often an awareness, but not a full understanding, of family planning indicators and their ability to accurately capture intended information, hampering the appropriate analyses.^25^ ^–^ ^27^ For example, when researchers in Tanzania asked study participants (e.g., family planning service providers, HMIS officers, district medical officers, facility in-charges) to identify the source of family planning indicator data, nearly 20% did not acknowledge men to be a source of family planning information, and one-third did not think any family planning data were obtained from youth.^27^

All the studies underscored that problems or perception of problems with data quality and reliability resulted in limited analysis and use of routine family planning data.

Many of the studies outlined mechanisms through which family planning data-capture tools might be used to improve data quality and thereby improve data analysis. Researchers in Tanzania recognized that incorporating explicit quality assessment indicators (such as quality of care or attitudes toward family planning) for family planning data into routine data collection could strengthen the usefulness of facility-level data when qualitative and quantitative indicators are analyzed together.^21^ The study authors added that an additional pathway for improved data quality and reliability was to explore and invest in technology options for data capture and transmission that were appropriate and cost-effective for rural settings and facilitated easier data analysis.^21^ In Nigeria, it was suggested that integrating family planning services in other health areas, such as HIV, immunizations, delivery, and postabortion care, could improve family planning data quality and reliability, and therefore analysis and interpretation, by creating a more complete picture of which family planning services are provided where and to whom.^13^

### Theme 4: Family Planning Data Use in Management, Programmatic, and Budgetary Decisions

The final theme identified in this review was family planning data use in management, programmatic, and budgetary decisions. Despite issues with data quality and reliability, routine family planning data were sometimes key for programmatic decision making.^26^ ^,^ ^27^ For example, in northern Tanzania, RHIS data were perceived to be an effective and important resource in decision making for improving family planning services.^26^ A member of a council health management team said^21^:

*RHIS is a very important tool to us in [council health management team]. We depend on it to make important decisions to improve health services in terms of understanding demand and resource allocation.*

However, the findings revealed that many management, programmatic, and budgetary decisions were not informed by evidence. For example, researchers in Nigeria found that despite the high unmet need for family planning (30.8%) in Cross River State, only 0.1% of the state’s health budget was earmarked for RH and family planning in 2014.^27^ (For comparison, in 2009–2010, RH represented 13.9% of total health expenditures in Kenya.^28^) In one case, the necessary data were not available; in Uganda, the National Medical Stores, development partners, and implementing partners were unable to access data on the quantity of family planning commodities imported and the cost price because the National Drug Authority did not have the data in retrievable form, even though organizations required this information for calculating budgets and funding needs.^29^ Use of the data for decision making often did not occur at lower levels of the system either.^15^ ^,^ ^25^

Findings revealed that many management, programmatic, and budgetary decisions were not informed by evidence.

Several factors limited capacity of information system users to analyze and use data in planning. In addition to issues discussed previously—such as the lack of training on the collection, analysis, and presentation of data or the lack of appropriate equipment to support data analysis—guidelines or systems were lacking on how to use routine data for decision making.^21^ ^,^ ^25^ ^,^ ^27^ In Tanzania, data use at the facility level was rare owing to a lack of perceived data ownership. Health providers expressed the belief that data could not be used at the point of creation and that they should only concern themselves with data collection.^25^ This finding was also seen in South Sudan and Nigeria, where data appeared to be used only to fulfill reporting requirements, not for analysis or decision making. To encourage data ownership and use at the facility level, one study recommended that supervisors at the district level provide regular feedback to facilities on their data, help facilities analyze the data for their needs, and give providers the opportunity to explain the data at meetings.^25^

Poor data quality was a barrier to data use for planning and budgeting in multiple studies.^18^ ^,^ ^25^ ^,^ ^26^ Tanzanian researchers found that data quality assurance, particularly accuracy, was a major challenge in the health facilities visited.^25^ In an in-depth interview, a service provider in Tanzania explained the consequences of poor data quality on decision making as follows^25^:

*In fact, the work plan is not realistic, there is a big difference between the work plan and budget. As you can see, this center is in the central part of the town. We serve more people than anticipated. For example, the budget has been prepared for 3,880 clients, but we serve 10,000 clients. We normally claim for the same, but they ignore us because we don’t have data. That’s why I say that there is a big difference between work plan and budget; the main reason for this is lack of correct data.* (Service provider at public health facility)

In South Sudan, researchers found that only one health facility included in the study made action-oriented decisions to mobilize or shift resources based on a comparison of services, and only one health facility made evidence-based decisions to advocate for more resources by showing gaps in its ability to meet monthly or annual targets.^15^ Several studies recommended in-service training to improve providers’ appreciation of how data could inform decisions and build capacity to analyze and use data.^14^ ^,^ ^15^ ^,^ ^25^ ^–^ ^27^

## DISCUSSION

The findings from the small grant–funded research reports provide an opportunity to identify specific examples of how information system challenges and shortfalls affect data quality and use. Similar to what has been reported in other countries, several small grant–funded studies revealed ongoing challenges with the technology and infrastructure necessary for electronic data collection and reporting.^30^ ^,^ ^31^ Although health service providers in multiple study countries expressed overall positive attitudes toward electronic data management and DHIS2, the lack of such basic inputs as providers trained in electronic data capture, a consistent power supply, reliable Internet connectivity, and a sufficient number of operative computers and mobile devices compromises the functionality of RHISs and the success of electronic HISs, including DHIS2. Such difficulties are not specific to family planning; they affect routine health information across all health areas.^32^ Government investments in these areas will improve the quality and utility of data infrastructure to strengthen the capacity of data management systems at health facilities.

Because many countries’ HISs have been strengthened to capture data on infectious diseases such as HIV, malaria, and tuberculosis, family planning appears to be an afterthought, with less attention and strategic planning for routine family planning data collection and use.^22^ The successful integration of family planning data in RHISs must accommodate data from disparate sources, ideally through standardized indicators and appropriate use of existing data collection tools along consistent operational guidelines. These tools include patient registries and reporting forms at the clinic, subnational, and national levels, among others. When data are not fully captured and aggregated from all family planning service delivery points and levels in the data management system—as the findings discussed here revealed—they provide an incomplete picture of the status of family planning service delivery and use in a given country. This situation in turn makes evidence-informed decision making difficult. The findings from the research projects pointed to several challenges with data collection tools (e.g., missing forms, incorrect versions, broken mobile devices, lack of guidelines for data collection), human resources (e.g., staff shortages, lack of data management training for personnel, absence of supportive supervision), and governance (e.g., lack of policies and guidelines for submission of data into the national HIS and lack of accountability mechanisms), which also affect data integration and compromise data quality.

Because many countries’ HISs have been strengthened to capture data on infectious diseases, family planning appears to be an afterthought.

Data quality, as defined by data accuracy, relevance, reliability, and timeliness, was found to be problematic in most of the small grant–funded research. Yet each of these characteristics is necessary to ensure integrity of data for policy and programmatic decision making.^1^ ^,^ ^8^ A common theme in the research studies was a lack of data training or solid understanding of the HIS and its potential for family planning data analysis and use. This translates into a lack of appreciation for complete, high-quality health data for decision making.

Data as a driver for decision making are integral to HIS performance and the improvement of health systems and outcomes; data use informs funding, policy, and national health goals.^1^ But if technical, management, organizational, financial, and political barriers to analyzing and using family planning data for planning purposes are present, as was demonstrated across several research studies, initiatives to improve the quality of family planning data will fail to achieve their potential. Fundamental changes in data culture will require strategies to motivate, mentor, and supervise staff at all levels, and staff must be included in programmatic reviews and decisions.

Data are integral to HIS performance and improved health systems and outcomes, and fundamental changes in data culture are needed at all levels.

### Strengths and Limitations

This synthesis presents the key findings from a body of research produced by local researchers in LMICs supported through MEASURE Evaluation’s 5-year small grants program. The synthesis provides access to research not available through peer-reviewed journals, highlighting context-specific findings from local researchers with specific insight on routine family planning data issues. The research findings have a unique focus on family planning in RHISs, and together provide information about RHISs that is relevant across systems and health areas and specific to the field of family planning. With a focus on routine data (i.e., service statistics), this synthesis identifies several areas for action and intervention to improve the functioning of RHISs and production of reliable, usable family planning information. The synthesis does not, however, attempt to present a comprehensive review of literature on RHISs or family planning information. Furthermore, the identification of key findings and the development of themes are based on the coauthors’ understanding and interpretation of the research. The authors acknowledge that the interconnected nature of routine data capture and production, reporting, analysis, and use make hard boundaries between themes difficult to define. The small grant-funded papers present additional detailed, context-specific research results.

## CONCLUSION

The breadth of the small grant-funded research papers revealed several opportunities and barriers related to the integration of family planning data in RHISs in LMICs and the countries’ ability to analyze and use the data to make programmatic and policy decisions. Lack of functioning electronic tools and resources in many contexts prevents providers from fully transitioning to an electronic HIS. A common theme among the study findings was poor data quality resulting from incomplete or missing data from private and nongovernmental organization facilities, insufficient or outdated data collection tools and forms, missing data collection guidelines, poorly defined indicators, and shortages of well-trained data-oriented service providers. Poor-quality data and a lack of data ownership, analysis skills, analysis tools, and a mandate and instruction from higher levels have prevented service providers from learning from their family planning data and making action-oriented decisions. The issues that contribute to poor data quality and its consequences are circular, self-reinforcing, and systemic. Addressing them requires long-term, multipronged interventions to improve family planning data management for well-informed decision making.

## Notes

**Peer Reviewed**

**First published online:** October 27, 2020
